# Evidence for a sex effect during overimitation: boys copy irrelevant modelled actions more than girls across cultures

**DOI:** 10.1098/rsos.170367

**Published:** 2017-12-06

**Authors:** Aurélien Frick, Fabrice Clément, Thibaud Gruber

**Affiliations:** 1 Department of Psychology, University of Edinburgh, Edinburgh, UK; 2 Laboratory of Experimental Psychology, Suor Orsola Benincasa University, Naples, Italy; 3 Cognitive Science Centre, University of Neuchâtel, Neuchâtel, Switzerland; 4 Swiss Center for Affective Sciences, University of Geneva, Geneva, Switzerland; 5 Department of Zoology, University of Oxford, Oxford, UK

**Keywords:** overimitation, innovation, tool-use, cross-cultural, sex differences, cumulative culture

## Abstract

Children are skilful at acquiring tool-using skills by faithfully copying relevant and irrelevant actions performed by others, but poor at innovating tools to solve problems. Five- to twelve-year-old urban French and rural Serbian children (*N* = 208) were exposed to a *Hook task*; a jar containing a reward in a bucket and a pipe cleaner as potential recovering tool material. In both countries, few children under the age of 10 made a hook from the pipe cleaner to retrieve the reward on their own. However, from five onward, the majority of unsuccessful children succeeded after seeing an adult model manufacturing a hook without completing the task. Additionally, a third of the children who observed a similar demonstration including an irrelevant action performed with a second object, a string, replicated this meaningless action. Children's difficulty with innovation and early capacity for overimitation thus do not depend on socio-economic background. Strikingly, we document a sex difference in overimitation across cultures, with boys engaging more in overimitation than girls, a finding that may result from differences regarding explorative tool-related behaviour. This male-biased sex effect sheds new light on our understanding of overimitation, and more generally, on how human tool culture evolved.

## 1. Introduction

Humans use sophisticated technologies daily. This remarkable technological advance results from our strong capacities for accumulating and transmitting cultural knowledge with improvements down generations [1], a process possibly requiring both high-fidelity imitation and innovation [2]. In recent years, much attention has been therefore devoted to understanding how these two features develop through childhood and how they compare to other animals, allowing for developmental, evolutionary and comparative perspectives [3].

With respect to high-fidelity imitation, young children selectively choose from whom and when to copy according to familiarity, age, trustworthiness of the model or experimental context [4,5]. Interestingly, contrary to chimpanzees (*Pan troglodytes*), children faithfully copy all actions, even those that are causally and explicitly irrelevant to success in a given task [6]. The absence of replication of causally irrelevant actions or ‘overimitation' [7] in other animals has been connected to limited evidence of cultural traits' accumulation across generations in non-human species (e.g. [2]), although the latter could also result from limitations in representational abilities [8].

In recent years, considerable efforts have been made to explain overimitation. Children may overimitate either because they misunderstand the causal status of irrelevant actions and automatically copy them (causal confusion account; [9]), because they desire to affiliate with the model (affiliative account; [10]), or because they interpret the irrelevant actions as an essential part of a bigger sequence of actions (normative account; [11]). Among these three reasons, the affiliative account may best explain why children overimitate less when the model is not present [12] or when the model is not an adult but a fellow child [13]. Similarly, the rational normative account provides better predictions regarding higher rates of overimitation once placed in the same context as the initial demonstration [14] or when the model performs an irrelevant action when relevant actions are more appropriate [15]. Interestingly, the normative account also predicts lower rates of overimitation when potential costs (e.g. time–pressure, bad consequences) or instrumental goals are associated with the irrelevant actions [16,17]. Taken together, these different experimental findings largely demonstrate that children are flexible overimitators.

However, a drawback to overimitation studies is that around 60% of them (based on our review at the time this article was written, see electronic supplementary material, table S1) have used the same puzzle box designed by Horner & Whiten [6]. In this paradigm, the model performs both relevant and irrelevant actions with the same tool (i.e. a stick) before retrieving a reward, questioning its application to real-life problems. As such, overimitation may have been elicited in these studies simply due to this paradigm drawing too much attention to the same single tool when acting on the puzzle box. Nevertheless, some studies have differed in several parameters when studying overimitation, for example, whether they relied on a tool or not, the tool material they used, the type of demonstrations or the task itself (e.g. [14–20]). In particular, Nielsen [20] used a naturalistic problem task, the *Floating Peanut task* [21], whereby the subject had to recover a reward by adding water to a tube to address both innovation and overimitation. Interestingly, unsuccessful children were presented with a model demonstration that did not lead to a clear result. This demonstration was aimed to be more realistic, as in many real-life situations we do not see end results of actions performed by others for various reasons (e.g. lack of time, attention distracted by something else, not enough visibility). Consistent with the behavioural re-enactment paradigm [22], which sees young children able to identify the aim of unfinished actions and complete them, Nielsen found that 4-year-old children faithfully copied all demonstrated actions, indicating that overimitation is a robust phenomenon. More recently, Taniguchi & Sanefuji [23] used a different puzzle box to investigate the effect of different action types on overimitation. They found that irrelevant actions were more replicated when these actions were directed towards the apparatus and involved the use of a tool, underlining that both the target of the action and the use of a tool contribute to enhance overimitation.

Similarly, in an attempt to characterize the influence of context (e.g. presence of the model, actions taking place in a different environment; [12,14]), Keupp *et al*. [14,17] investigated overimitation in series of studies relying on a variety of puzzle games. Interestingly, the authors did not find lower overimitation rates in games in which the irrelevant and relevant actions were performed by two separate tools (e.g. Task B, [14]). A separate tool used for irrelevant actions was selectively as much interpreted as normative as a single tool used for both irrelevant and relevant actions, suggesting the absence of a tool-confound. However, it is unclear how this finding applies to (i) a naturalistic problem-solving context, where (ii) one tool is used in relevant actions and another one in irrelevant actions, and (iii) after an initial failure to figure out the solution by the participant. For example, in a situation in which children can easily identify the correct tool but fail to understand how it can be used to solve the problem, to what extent would they then copy the irrelevant actions performed with another separate tool they easily identify as not being part of the solution? This question remains entirely open, and appears of importance to understand how children engage in overimitation in real-life situations.

Comparatively, innovation in children has received attention only recently despite being widely investigated in non-human animals (see [24]). Beck *et al.* [25] presented 3- to 7-year-old children with a child-adapted version of the *Hook task* [26], where subjects had to manufacture a hook out of a straight piece of material to retrieve a reward. Surprisingly, children started retrieving the reward by bending the pipe cleaner successfully only from 9 years old. Younger children experienced similar difficulties to innovate even when they could manipulate the material beforehand and were explicitly told to ‘make something' with the material, or in other tool-making tasks (e.g. [20,25,27,28]). These observations strongly contrast with children's precocious imitative abilities and seem at odds with the hypothesis that social learning and asocial learning abilities correlate with each other [29], questioning how these two abilities interacted to lead to the human cultural phenomenon [4].

But do the studies described above give an accurate picture of human development in general, rather than development in a particular social and economic context? In a recent cross-cultural study, Berl & Hewlett [30] showed that children without formal schooling engaged in overimitation only from the age of seven, suggesting possible variations in what has been assumed to be an otherwise universal phenomenon [31–33]. Similarly, the wide availability of manufactured tools in privileged societies has been proposed to explain the limited performance of children in innovation tasks [34] although the only cross-cultural study on the topic so far has failed to confirm this claim [35] and no other study has attempted to compare populations of different socio-economic backgrounds.

Here, following Nielsen [20], we aimed to study both overimitation and innovation within one task, the *Hook task*. We also aimed to disentangle overimitation from the confound of using one single object in the relevant and irrelevant actions by using separate tools for the two actions in a situation during which children would experience failure in a naturalistic problem-solving task. We first observed whether children succeeded in recovering a reward by manufacturing a hook. If they did not succeed, they were randomly exposed to one of two demonstrations: either only a relevant action using a pipe cleaner; or the relevant action using a pipe cleaner preceded by an additional irrelevant action using a string. We then examined whether additional factors such as age, sex or socio-economic variations affected innovation and overimitation. In particular, we explored these two behaviours in typical young children living either in a French urban city or in a Serbian rural town and village, giving our study a cross-cultural dimension. Indeed, while it is debatable whether France and Serbia can both be equally categorized as ‘WEIRD' countries (Western, Educated, Industrialized, Rich and Democratic) according to Henrich *et al.* [36], there are several important socio-economic differences between the two countries. For instance, even though the Serbian population has access to formal schooling, the quality of the Serbian educational system has been described as lower than in most developed countries, resulting in a high secondary education dropout rate [37]. Furthermore, the rising price of manufactured products coupled with a weak average monthly salary ($400) and a high level of unemployment result in lower living standards for Serbian families, particularly in rural environments, when compared to French families [38]. As an example, only half of rural Serbian households are connected to the Internet (53.9%) compared with 83.5% of connected French households [38,39]. Because of these differences in socio-economic factors, children from rural Serbia offered an opportunity to test the universality of characteristics of innovation and overimitation. Regarding innovation, we were interested in retesting the hypothesis that children from underprivileged environments who have limited access to manufactured toys may rely more on imagination and creativity when playing, a hypothesis which was not confirmed in the few experimental tasks that addressed it [35,40]. Regarding overimitation, as both French and Serbian children receive formal education but with some apparent variations in terms of quality, our comparison would also allow evaluating whether variations in this particular form of teaching influenced overimitation rates across cultures [30]. All in all, a cross-cultural comparison between French and Serbian children would thus extend previous different cross-cultural studies on the developmental trends of innovation and overimitation, allowing assessing the presupposed universality of innovation and overimitation in children in an understudied additional culture.

We predicted that innovation would be difficult for children up until late childhood and that this would contrast with an early capacity for imitation [4,25]. In particular, we expected that unsuccessful children presented only with a relevant action that did not lead to a clear outcome would be able to infer the goal and the functionality of the action to retrieve the reward out of the bottle [20]. Similarly, we predicted that unsuccessful children assigned to the Overimitation condition would be able to infer the goal and the functionality of the action to retrieve the reward; however, we also expected them to increasingly reproduce the irrelevant action with age [41]. Given the instrumental nature of our task, we also expected to find lower rates of overimitation in comparison to what is generally found in the literature [17]. Finally, because of the suspected universality of the phenomenon [30–33,35], we did not expect differences regarding innovation, imitation or overimitation rates between French and Serbian children.

## 2. Material and methods

### 2.1. Participants

**Figure 1.**
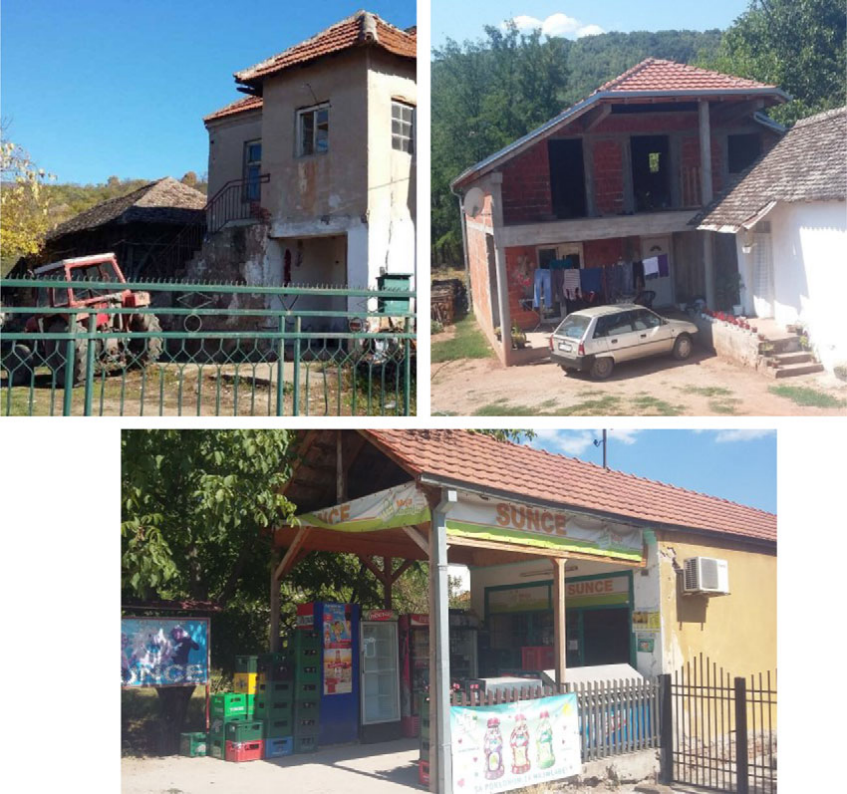
Picture of two typical houses and the minimarket in the rural Serbian village (©Aurélien Frick).

Participants were typical French children attending private schools located in a medium-sized (approx. 115 000 inhabitants) urban city in the centre of France, and rural Serbian children attending public schools in a small-sized rural town (approx. 12 000 inhabitants) and a small rural village (approx. 300 inhabitants) in eastern Serbia. The rural Serbian town and village were separated by 10 km and were located 40 km away from the third largest city in Serbia, Niš (approx. 260 000 inhabitants). In Serbia, the population lived in modest houses surrounded by few restaurants and minimarkets selling few items (e.g. small car toys) for children (figure 1). The village had one small school educating around 40 children, aged between 6 and 10 years old taught in age-mixed classrooms. There were two schools in the town, one educating about 70 children from 3 to 6 years old and divided into two institutions, and the other about 130 children from 11 to 15 years old. In comparison, the three French schools located in the same city educated 175 children from 3 to 6 years old, 83 children from 3 to 6 years old, 176 children from 6 to 11 years old, and 144 children from 6 to 11 years old. In both cultural groups, children were grouped and taught in similar age classes (except for the school in the Serbian village).

A total of 236 French and Serbian children aged between 4 and 12 years old were tested. From this sample, eight Serbian children were first excluded as their demographic information was missing, which led to a sample of 228 children (108 boys and 120 girls, *M*_age_ = 8.39 years, SD = 2.15 years, age range = 4.33–12.33 years). We then removed 10 children who did not use any tools during the pre-demonstration phase and/or the post-demonstration phase despite the second prompt by the experimenter (one French child and nine Serbian children); one French child who succeeded in retrieving the reward by chance; and one additional Serbian child due to an experimental error. We also excluded 4-year-old children because there were only eight of them (five French children and three Serbian children). Our final sample was therefore composed of 208 French and Serbian children aged 5 to 12 years old (98 boys and 110 girls, *M*_age_ = 8.66, s.d. = 2.00, age range = 5.00–12.33) with 102 French children (47 boys and 55 girls, *M*_age_ = 8.61, s.d. = 2.08, age range = 5.17–12.33) and 106 Serbian children (51 boys and 55 girls, *M*_age_ = 8.71, s.d. = 1.94, age range = 5.00–12.25). There was no significant difference between French and Serbian children regarding their age (France: *M_d_* = 8.29; Serbia: *M_d_* = 8.83; *U* = 5252.50, *p* = 0.724), and sex (France: 47 boys, 55 girls; Serbian: 51 boys, 55 girls; *χ*^2^ (1, *N* = 208) = 0.09, *p* = 0.769, *φ_c_* = 0.02).

### 2.2. Materials and procedure

All children were tested individually in a quiet space within the school but in isolation from other children. A male French native speaker and two female Serbian native-speaking research assistants conducted the experimental sessions, in France and Serbia, respectively. Each experimental session began with a Warm-up task to put children at ease with the experimenter and was followed by the *Hook task*. In the Warm-up task, children were shown four laminated cards (12.5 × 6.5 cm), half of them displaying four animal drawings and the other half, four fruit drawings. Two cards were presented to them, either in the animal–fruit or fruit–animal order, and the children were asked to name each element. Subsequently, children were shown the two remaining cards in the same order (animal–fruit or fruit–animal) and asked to name the element, which had been on both cards.

**Figure 2.**
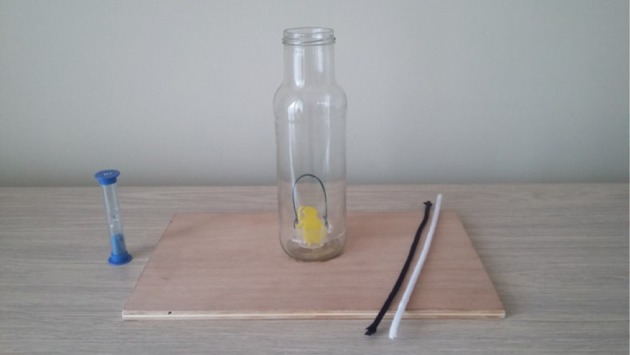
Material used for the Hook task.

In the *Hook task*, as in previous developmental studies on innovation, children were presented with a transparent glass jar (height = 23 cm, width of opening = 4 cm) attached to a wooden base (length = 35 cm, width = 21 cm) containing a bucket (height = 7.5 cm, width = 3 cm) with a reward inside (figure 2). They were informed that their task was to retrieve the reward and that if they succeeded in doing so, they would be allowed to keep it. Subsequently, the experimenter displayed on the table a black/white straight pipe cleaner and a black/white piece of string (length = 30 cm and colour counterbalanced) and then prompted the participants by saying ‘You may use these things to help you to retrieve the little animal out of the bottle'. The children were given a minute, measured with a visible sand-timer, to retrieve the reward. If children did not use or enter any tool in the bottle after about 30 s, they were prompted ‘Do not forget that you can use these things to retrieve the little animal out of the bottle’ by the experimenter.

**Figure 3.**
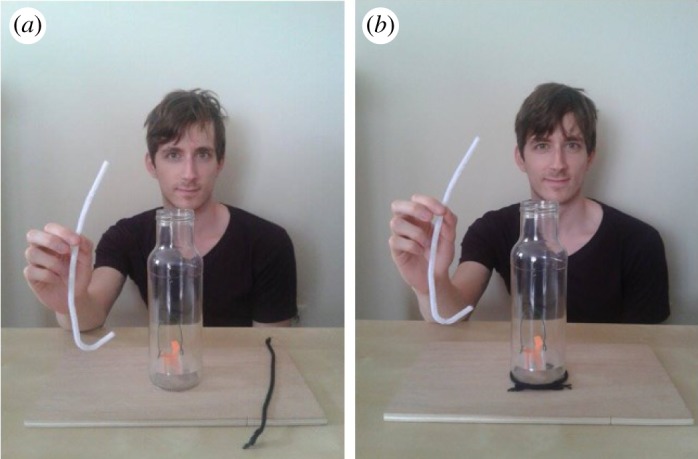
End-state of the demonstration in the Control condition (*a*) and in the Overimitation condition (*b*).

For the children who successfully retrieved the bucket out of the bottle with a functional pipe cleaner within a minute, the experiment stopped. They were told that they could keep the reward and that the experiment was finished. As such, they were not assigned to any demonstration condition. The children who did not successfully retrieve the reward by the end of the time limit were randomly assigned either to the Control condition or to the Overimitation condition. In both conditions, the experimenter first told them to release the material they were holding; she/he then displayed a new straight pipe cleaner and a piece of string on the table while saying ‘Watch'. Children assigned to the Control condition saw the experimenter seize the pipe directly on the middle and make a hook at the bottom extremity. Children assigned to the Overimitation condition observed the experimenter first circling the bottle with the string before making a hook with the pipe cleaner (figure 3). After the demonstration, the experimenter said ‘Now can you try to retrieve the reward out of the bottle?' and remained present beside the children after having demonstrated the action(s).

### 2.3. Coding

For the pre-demonstration phase, we coded whether or not children successfully retrieved the reward out of the bottle by manufacturing (innovating) a hook using a pipe cleaner. The same behaviour was reported for the post-demonstration phase for all children sampled. Children assigned to the Overimitation condition were considered as overimitators if they first used the string to make a full and closed circle around the bottle before manipulating the pipe cleaner. All the videotapes were coded by the first author; a second coder, blind to the objectives of the experiment, independently double coded 30% of the recordings (62 out of 208) with *κ* = 1 for each measure.

### 2.4. Data analysis

To analyse our data, we conducted binary logistic linear regressions using SPSS 21.0 [42]. For the pre-demonstration condition, the model included main effects and interactions between the following factors: ‘age' (in months), ‘culture' (French versus Serbian) and ‘sex' (boy versus girl) on the categorical dichotomous-dependent variable of ‘success’ (yes versus no). For the post-demonstration condition, the model included main effects and interactions between the same factors with an additional ‘condition' (control versus overimitation) on the same variable of ‘success’. We also ran a model for the Overimitation condition that included main effects and interactions between the factors ‘age', ‘culture' and ‘sex' on the variable ‘overimitation' (yes versus no). Importantly, we first tested models with all possible interactions (all included in electronic supplementary material, tables S2–S4). However, even though these models were significantly different from the null model, no interaction was significant. We therefore removed the interactions from the models, ran them again and used models without interactions for the presentation of the results. Finally, we used a continuous variable for the factor ‘age' to avoid any loss of information when running the model, but when we found an important effect of this factor we subsequently used *χ*^2^ tests to make adjacent comparisons regarding 2-year window age groups. More specifically, we categorized children into four age groups (5–6 years old, 7–8 years old, 9–10 years old and 11–12 years old) corresponding to the usual age division per classes in French and Serbian schools, making also possible to have sufficient statistical power and/or roughly the same number of children in each group (except for the older group that was composed of only 33 children; for more details on these age groups, see electronic supplementary material, table S6).

## 3. Results

### 3.1. Pre-demonstration phase—innovation

**Figure 4.**
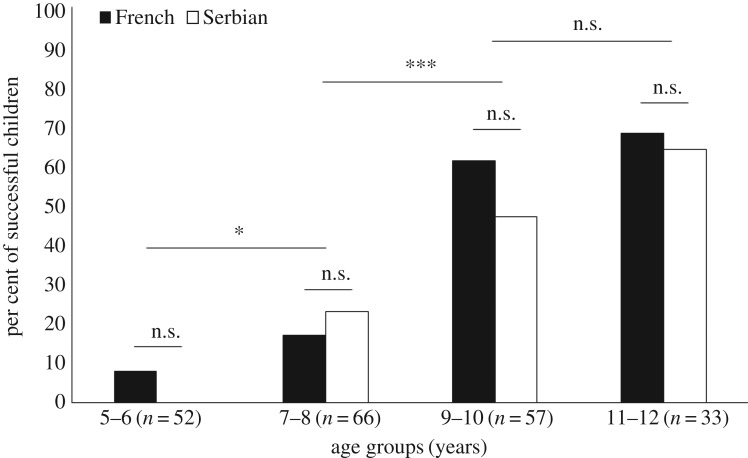
Percentage of successful children in the pre-demonstration phase as a function of four different age groups and cultural background. *p < 0.05 and ***p < 0.001.

In the pre-demonstration phase, only 67 out of 208 (32%) children innovated a functional hook with the pipe cleaner and successfully retrieved the reward with it (details on behaviours observed in the pre-demonstration phase are included in electronic supplementary material, table S7). We ran a binary logistic linear regression model to explore the effects of age (in months), cultural background (French versus Serbian), sex (boy versus girl) on the variable ‘success’ (table 1). This model was statistically significant ($\chi^{2}(3)=58.60$, *p *< 0.001), explained 34.30% of the variance (Nagelkerke *R*^2^), correctly classified 76.00% of cases, and indicated a strong positive effect of age (*B* = 0.06, *p* < 0.001). That is, older children manufactured a functional hook significantly more than younger children. We further explored this age effect within four age groups with a 2-year window (for details on these age groups, see electronic supplementary material, table S2). As illustrated in figure 4, comparisons of adjacent age groups indicated that 7- to 8-year-old children were significantly more likely to bend the pipe cleaner than younger children (*χ*^2^ (1, *n* = 118) = 6.59, *p* = 0.010, *φ_c_* = 0.24), but they were less likely to do so than those aged 9 to 10 years old (*χ*^2^ (1, *n* = 123) = 14.59, *p* < 0.001, *φ_c_* = 0.34). By contrast, although more 11- to 12-year-olds than 9- to 10-year-olds bent the pipe cleaner, this did not reach statistical significance (*χ*^2^ (1, *n* = 90) = 1.69, *p* = 0.194, *φ_c_* = 0.14).

**Table 1.** Binary logistic linear regression—success during the pre-demonstration phase. *R*^2^ = 0.34 (Nagelkerke); correct classified cases = 76.00%; model *χ*^2^ (3) = 58.60, *p* < 0.001.

predictor	*B*	s.e.	Wald	d.f.	*p*	odd ratio
age	0.06	0.01	40.89	1	<0.001	1.06
cultural background	0.32	0.35	0.86	1	0.353	1.38
sex	−0.26	0.35	0.56	1	0.456	0.77

### 3.2. Post-demonstration phase—success

**Figure 5.**
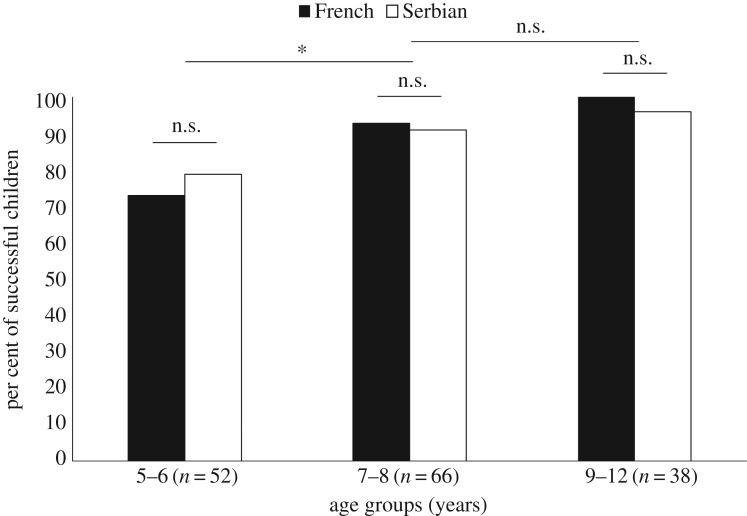
Percentage of successful children in the post-demonstration phase as a function of three different age groups and cultural background. *p < 0.05.

In the post-demonstration phase, that is after being shown how to make a hook with the pipe cleaner, most of the children successfully retrieved the reward (124 out of 141, 88%, see electronic supplementary material, table S10). We conducted a binary logistic linear regression model exploring the effect of age, cultural background, sex, condition (control versus overimitation) on the variable ‘success’ ($\chi^{2}(4)=18.49$, *p* = 0.001, *R*^2^ = 0.24 (Nagelkerke); correctly classified cases = 88.70%). This model indicated a positive effect of age (*B* = 0.07 *p* = 0.001, table 2) but no significant effect of cultural background (*B* = 0.30, *p* = 0.598), condition (*B* = 0.86, *p* = 0.153) or sex (*B* = 0.04, *p* = 0.947). As such, in both conditions and cultures older children were significantly more likely to successfully retrieve the reward after observing how to make a functional hook than younger children. To make comparable age group comparisons, children were grouped into three different groups instead of four. Indeed, because few 11--12-years-olds (*n* = 11) were assigned to one of the two demonstrations, they were grouped with the 9–10-year-old category (*n* *=* 27) to have more comparable categories in terms of number of children (5–6 year old: *n* = 50; 7–8 year old: *n* = 53; 9–12 year old: *n* = 38, for more details, see electronic supplementary material, table S9). As illustrated in figure 5, inter-age group comparisons revealed that the only statistical difference was between the 5- to 6-years-olds and the 7- to 8-year-olds (*χ*^2^ (1, *n* = 103) = 5.31, *p* = 0.021, *φ_c_* = 0.227) even though the level of success was particularly high for all age groups (electronic supplementary material, table S10).

**Table 2.** Binary logistic linear regression—success during the post-demonstration phase. *R*^2^ = 0.24 (Nagelkerke); correct classified cases = 88.70%, model *χ*^2^ (4) = 18.49, *p* = 0.001.

predictor	*B*	s.e.	Wald	d.f.	*p*	odds ratio
age	0.07	0.02	12.06	1	0.001	1.07
cultural background	0.30	0.57	0.28	1	0.598	1.35
sex	0.04	0.56	0.004	1	0.947	1.04
condition	0.86	0.61	2.04	1	0.153	2.37

### 3.3. Post-demonstration phase—control versus overimitation

**Figure 6.**
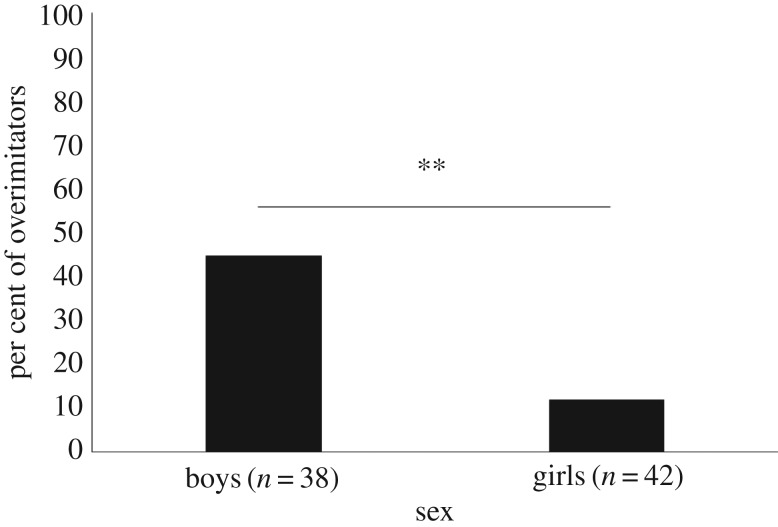
Percentage of overimitators as a function of sex. **p < 0.01.

With regard to the presence of a second irrelevant tool, only 1 out of the 61 children assigned to the Control condition touched or used the string, which significantly contrasted with those assigned to the Overimitation condition (25 out of 80, 31%, electronic supplementary material, table S10). Among the children touching or using the string in the Overimitation condition, 22 out of the 25 children copied the irrelevant action performed by the model (28% of 80 participants, see electronic supplementary material, table S11). A binary logistic regression model ($\chi^{2}(3)=12.23$ , *p* = 0.007; *R*^2^ = 0.20 (Nagelkerke); correctly classified cases = 72.5%, table 3) investigating the effect of age, cultural background and sex on the variable ‘overimitation' found a strong negative effect of sex (*B* = −1.77, *p* *=* 0.003) but no effect from other factors. Boys were significantly more likely to overimitate than girls, with about half of the boys tested (17 out of 38) but only 12% of the girls tested (5 out of 42) engaging in overimitation (*χ*^2^ (1, *n* = 80) = 10.79, *p* < 0.001, *φ_c_* = 0.38, figure 6).

**Table 3.** Binary logistic linear regression—circle the bottle with the string in the overimitation condition. *R*^2^ = 0.20 (Nagelkerke); correct classified cases = 72.5%, Model *χ*^2^ (3) = 12.23, *p* = 0.007.

predictor	*B*	s.e.	Wald	d.f.	*p*	odds ratio
age	0.01	0.01	0.76	1	0.38	1.01
cultural background	0.33	0.55	0.36	1	0.55	1.39
sex	−1.77	0.59	9.03	1	0.003	0.17

**Figure 7.**
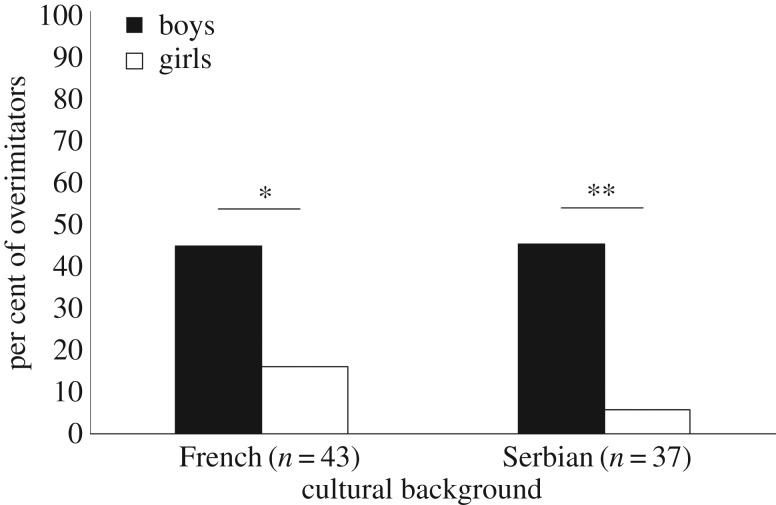
Percentage of overimitators as a function of sex and cultural background. *p < 0.05; **p < 0.01.

Additionally, the male-bias was found both in French and Serbian children (figure 7). For French children, 8 out of 18 boys (44.45%) engaged in overimitation compared to 4 out of 25 girls (16%, *χ*^2^ (1, *n* = 43) = 4.21, *p* = 0.040, *φ_c_* = 0.31). For Serbian children, 9 out of 20 (45%) boys engaged in overimitation, compared to 1 out of 17 girls (5.88%, *χ*^2^ (1, *n* = 37) = 7.13, *p* = 0.008, *φ_c_* = 0.44).

## 4. Discussion

Consistent with previous studies, most children below the age of 10 years old did not succeed in manufacturing a new functional tool to retrieve the reward. Critically, both cultural groups were similarly impaired, confirming that socio-economic environment does not affect innovative abilities. Also consistent across cultures, a majority of unsuccessful children from 5 years old and older manufactured and used a functional hook to retrieve the reward after witnessing a demonstration. However, we obtained a much smaller rate of overimitation (less than 30%) compared to most other studies, and found an unexpected consistent sex effect in both populations with boys much more likely to engage in overimitation than girls. This finding appears of importance because few overimitation studies have reported sex differences: it is unclear whether this is because sex was not investigated as a variable as ‘[…] there [was] no prior reason to suspect gender effects’ in overimitation [43, p. 198], or because no sex effect was found when investigated (60% of the studies reviewed in electronic supplementary material, table S1 did not mention any sex variable in their results, and, when considered (40%), none reported significant effects without any confounding variables such as the sex of the model [44]). We discuss each result individually in the following.

Our results demonstrate firstly that young children, while limited in their innovative abilities [27], remain particularly skilful at socially acquiring tool-use knowledge from others across cultures [45]. However, because all tested children attended formal schools and there was no clear difference between Serbian and French children, it is still unclear whether formal teaching may affect innovative skills when learning how to solve new problems [35]. To address this point, future investigations should therefore consider children who experience less formal teaching, such as those attending the Montessori pedagogy or children raised in hunter–gatherer societies; with the prediction that they may outperform typical children because they usually rely more on themselves when faced with such problems and have been shown to display better abilities for self-regulation and creativity [46]. Nevertheless, the effect of other variables that may differ between children, e.g. parenting style, should also be investigated. Beyond cultural differences, because our demonstrations highlighted how to manufacture a functional hook without showing what could be done with it, our results also confirm that young children, regardless of their social upbringing, are good at inferring goals underpinning actions performed on objects that do not lead to a clear outcome; and at reproducing the desired outcome of these actions [19].

With regard to overimitation, the fact that less than a half of the children copied the irrelevant action is in line with recent studies emphasizing that overimitation in children is not automatic (e.g. [14,17]). In particular, a rational normative evaluation of the necessity to replicate actions (e.g. [11]) appears consistent with our results. Indeed, most children may have refrained from overimitating because they did not interpret the irrelevant action as normative due to the use of different objects in the two actions during the demonstration. The use of different tools to carryout the irrelevant and relevant actions could have increased the salience of the distinction between these actions to the children, leading them to interpret the irrelevant action as being genuinely irrelevant, and possibly not normative [43]. Furthermore, this result may have been increased by the problem-solving context in which the *Hook task* was embedded. For example, while observing the demonstration, children often verbalized ‘Oh yes!' when the experimenter bent the pipe cleaner. As such, when they attempted to retrieve the reward they may have focused more on correctly bending the pipe cleaner to complete the task rather than on the irrelevant action involving the string. However, the limited rate of overimitation in our study genuinely contrasts with the high rates observed in the few studies that have disentangled overimitation from the tool-confound ([14,17]; see electronic supplementary material, table S1). Similarly, our results do not seem to follow studies showing that the presence of an expert model (here, the experimenter) leads to higher rates of overimitation as it increases the conventional aspect of the different actions performed [41,44]. Rather, the instrumental nature of our task (i.e. the focus being on achieving an instrumental goal, and more specifically on retrieving the reward out of the bottle) may better explain why so few children copied the irrelevant action. In a recent study, Keupp *et al*. [17] have indeed shown that when instructions are instrumentally framed (‘I will show you one way to get the toy out', p. 39) rather than conventionally framed (‘I will show you how to get the toy out', p. 39), the rate of overimitation is significantly lower. In our study, after the demonstration, the sentence we used to prompt the children (‘Now can you try to retrieve the reward out of the bottle?’) can be considered an instrumentally framed instruction, which may have led most children to focus on the relevant tool and action to get the reward out of the bottle.

Beside this possible ‘prioritizing efficiency over overimitation' strategy based on a rational normative interpretation, our data may also be explained through a causal explanatory account [7,9]. After having seen the demonstration, children may have indeed attributed different goals to the actions based on whether the irrelevant and relevant actions were new or old for them (e.g. [47,48]). Thus, it might be that most of our unsuccessful children did not replicate the irrelevant action because when faced with two items of novel information (i.e. bending the pipe cleaner and circling the bottle with the string), they prioritized the goal-relevant action over the goal-irrelevant action as the instrumental goal of the experimenter was to retrieve the reward out of the bottle [48]. Conversely, successful children for whom the only new information would have been the irrelevant action might have assigned higher relevance to this action and be, therefore, more prone to replicate it. Unfortunately, a caveat of our experimental design was that we did not present our successful children with a demonstration; as a consequence we could not test this assumption. Therefore, an interesting avenue for future research would be to assign both successful and unsuccessful children in the pre-demonstration phase to the post-demonstration conditions, bringing potentially new information about how task understanding may lead children to behave differently in social learning and more particularly overimitation tasks. This would possibly also raise the percentage of children engaging in overimitation; bringing it closer to traditionally recorded values in the literature (see electronic supplementary material, table S1).

Our most striking and unexpected finding, however, is the potent male-bias observed across cultures during overimitation. To our knowledge and following a review of the literature, this is the first time that such a sex bias has been observed (see electronic supplementary material, table S1). Previous studies have reported that children prefer to endorse information coming from informants of their own sex [49], which may eventually result in sex differences when it comes to overimitation in adults: women have indeed been observed to copy more precisely and more rapidly irrelevant tool actions performed by male models than female models [50]. However, in our study the Serbian children were all tested by female experimenters and showed the same sex bias as the French children (tested by one male experimenter), thus the sex of the model is unlikely to have played a role in our results. This male-bias also appears inconsistent with sex differences found in conformity tasks. Indeed, no sex difference has been reported when children conform to adults [51], the only difference appearing when the model is a peer, with girls conforming more than boys [5,52], an effect persisting in adulthood [53]. Thus, a same-sex or conformity effect may not explain our results. Rather, a first possibility is that this sex bias could be explained by an unequal distribution of children across the two conditions. Indeed, as illustrated in electronic supplementary material, table S10, there were more girls than boys in the two youngest age groups (5–6: 11 boys and 13 girls; 7–8: 14 boys and 20 girls), while there were more boys than girls in the older group (9–12: 14 boys and 7 girls). Given that overimitation increases with age [41], it could, therefore, be argued that our sex effect could be due to the fact that a larger number of older boys were assigned to the Overimitation condition. First, we did not find any significant effect of the interaction between age and sex, which undermines this possibility (electronic supplementary material, table S4). Additionally, looking at our data more closely, we find that the sex effect appears in the 7- to 8-year-old group (7 out of 14 boys and 4 out of 20 girls) in which there were more girls than boys. This indicates that although our samples were particularly small in the older age groups due to the specificity of our methodology and somehow unequally distributed, the boy-biased sex effect remained stable in all age categories.

Rather, we hypothesize that the difference between boys and girls regarding overimitation here may result from the presence of several objects coupled with the specific situation we created: the use of a naturalist problem-solving task and experiencing a failure before engaging in the task again. Our hypothesis builds more specifically on sex differences found in humans and apes for tool use. Indeed, in human children, boys use more tools when solving a problem, and increasingly consider more objects in their environment as potential instrumental tools than girls [54]. Such male-bias regarding object manipulation has been recently reported in infant chimpanzees [55], compared to a general female-bias observed in tool-use for mature individuals in the *Pan* genus [56]. Given this putative male-bias in tool-exploration and the fact that the demonstration involved several tools in an instrumental task, it might be that boys overimitated more because they paid more attention to all objects at their disposal than girls, increasing their likelihood to engage with the string. Whether this also resulted in facilitating a rational normative interpretation of the irrelevant action or in inferring different goals according to the novelty of the relevant and irrelevant action is another question that will need to be explored. Nevertheless, the sex factor, along with other intrinsic characteristics of the participant (e.g. personality) and the task itself, may be a new factor interacting with the normative interpretation or causal knowledge dimensions to produce overimitation. As such, future work should more systematically investigate intrinsic characteristics of participants in order to better understand how overimitation occurs.

## Supplementary Material

Literature review of overimitation studies, descriptive results and statistical models

## Data accessibility

The data that support the findings of this study are available through the Open Science Framework at https://osf.io/2e86d [57].
