# Supplementary material for: Evidence for a sex effect during overimitation: boys copy irrelevant modelled actions more than girls across cultures
Source: R Soc Open Sci. 2017 Dec 6;4(12):170367. doi: 10.1098/rsos.170367 (PMC5749984; doi:10.1098/rsos.170367)
Supplement: Literature review of overimitation studies, descriptive results and statistical models [file rsos170367supp1.doc]

**Evidence of a sex effect during overimitation: Boys copy irrelevant modelled actions more than girls across cultures**

Aurélien Frick, Fabrice Clément and Thibaud Gruber

**Supplementary Information**

- Table S1: Overview of studies on overimitation
- Table S2: Binary logistic linear regression - Success (Pre-demonstration phase) – With all interactions
- Table S3: Binary logistic linear regression – Success (Post-demonstration) – With all interactions
- Table S4: Binary logistic linear regression - Over-imitation – With all interactions
- Table S5: Binary logistic linear regression – Success (Overimitation condition) – With all interactions
- Table S6: Binary logistic linear regression – Success (Overimitation condition)
- Table S7: Demographic information regarding (a) the final sample, (b) the French sample and (b) the Serbian sample for the pre-demonstration phase
- Table S8: Summary of behaviours observed (frequencies and percentage) in the pre-demonstration phase as a function of age groups and cultural background
- Table S9: Demographic information regarding (a) the final sample, (b) the French sample and (b) the Serbian sample during the post-demonstration phase (Control and Overimitation condition)
- Table S10: Frequencies and percentage of touching/using the string and success in the post-demonstration as a function of age groups, cultural background and conditions
- Table S11: Frequencies and percentage of overimitators and emulators as a function of age groups, cultural background and Sex

**Table S1: Overview of studies on overimitation**

Studies presented below are referenced in the chronological order, * indicates that the same puzzle boxes than the initial study by Horner and Whiten (1) involving a single tool to perform the relevant and irrelevant actions were used and † indicates cross-cultural comparisons. When possible, results are precisely those indicated in the study, otherwise indicative approximations based on the graphs available are given.

For the sex variable (i.e., the column ‘Sex differences’), we first looked at whether this variable had been tested in the studies. If the sex variable was tested, we report whether any difference was found or not (‘Yes’ or ‘No’). If ‘Yes’, we also indicate in which direction an effect was observed. If the sex variable was not tested and did not appear in the target paper as a control variable, we indicate ‘Not indicated’.

Among the 30 studies reviewed in the table below, 12 (40%) mentioned a gender/sex variable in their analyses and 11 (92%) did not find any effect. The only study finding sex/gender effects is the study by McGuigan (2013), indicating that women overimitated more than men, an effect due the confounding variable of the model’s sex.

| **Study** | | **Sample size** | **Sex ratio** | | | **Age range** | | **Task(s)** | **Tool(s)** | | | **Causal relevance (Rel) and irrelevance (Irrel) of action** | | **Overimitation ratio** | **Sex differences** | |
| --- | --- | --- | --- | --- | --- | --- | --- | --- | --- | --- | --- | --- | --- | --- | --- | --- |
| **Horner and Whiten (1), Experiment 4** | | 16 | 8 M, 8 F | | | 41-59 m/o | | Clear puzzle box  Opaque puzzle box | Stick | | | Stick in Rel and Irrel action | | **% tool insertions into**  **top irrelevant hole**  Group A/B: clear: 80, opaque: 70.84%  Group C/D: clear: 75, opaque: 80 | No | |
| ***Lyons, Young (2), Experiment 1A and 1B** | | 63 | Not indicated | | | 3-5 y/o | | Clear puzzle box  Cage puzzle  Dome puzzle | Wand  Puzzle mechanism | | | Wand in Rel and Irrel action  Hand in Rel action and side or wooden handle in Irrel action | | **% children copying the irrelevant action in each box**  Clear puzzle box: 95%  Cage puzzle: 50%  Dome puzzle: 80%  **% children copying the irrelevant action in each box after contrary pressure**  Clear puzzle box: 50%  Cage puzzle:40%  Dome puzzle: 50% | Not indicated | |
| ***Lyons, Young (2), Experiment 2A** | | 29 | Not indicated | | | 3-5 y/o | | Clear puzzle box  Dome puzzle | Wand  Puzzle mechanism | | | Wand in Rel and Irrel action  Hand in Rel action and side or wooden handle in Irrel action | | **% children copying the irrelevant action despite warning**  Clear puzzle box: 75%  Cage puzzle:70% | Not indicated | |
| **Lyons, Young (2), Experiment 2B** | | 29 | Not indicated | | | 3-5 y/o | | Igloo connected  Igloo disconnected | Puzzle mechanism | | | Hand in Rel action and action on wooden wand in Irrel action | | **% children copying the irrelevant action despite warning**  Igloo connected: 55%  Igloo disconnected: 20% | Not indicated | |
| ***McGuigan, Whiten (3)** | | 48  48 | 21 M, 27 F  24 M, 24 F | | | 3 y/o  5 y/o | | Clear puzzle box  Opaque puzzle box | Stick | | | Stick in Rel and Irrel action | | **Irrelevant imitation score (max 1)**  3 y/o: live: .59, video: .17  5 y/o: live: .67, video: .55 | Not indicated | |
| ***McGuigan and Whiten (4)** | | 24  24 | Total  22 M, 26 F | | | 18-24 m/o  26-35 m/o | | Clear puzzle box  Opaque puzzle box | Stick | | | Stick in Rel and Irrel action | | **Irrelevant imitation score (max 1)**  23 m/o: clear: .25, opaque: .10  30 m/o: clear: .30, opaque: .12, | Not indicated | |
| *****†**Nielsen and Tomaselli (5), Experiment 1** | | 16 Brisbane  6 Witdraai  10 Ngwatle | 9 M, 7 F  3 M, 3 F  3 M, 7 F | | | 2-6 y/o | | Blue box  Switch box  Opaque puzzle box | Red stick  Mallet  Blue stick | | | Stick and mallet in Rel and Irrel action | | **% children copying the irrelevant action in each box**  Brisbane and Bushman (Witdraai and Ngwatle): 75% | Not indicated | |
| **Nielsen and Tomaselli (5), Experiment 2** | | 62 Westerners | 29 M, 33 F | | | 2-13 y/o | | Blue box  Switch box  Opaque puzzle box | Red stick  Mallet  Blue stick | | | Stick and mallet in Rel and Irrel action | | **Number of boxes in which children produce the irrelevant action**  Brisbane and Bushman (Witdraai and Ngwatle): 2.7, Westerners: 2.8 | Not indicated | |
| ***McGuigan and Graham (6)** | | 32  32 | Not indicated | | | 3 y/o  5 y/o | | Clear puzzle box  Opaque puzzle box | Stick | | | Stick in Rel and Irrel actions | | **% of children copying irrelevant actions**  3 y/o: clear: 100%, opaque: 100%  5 y/o: clear: 14%, opaque: 100% | No | |
| ***Kenward, Karlsson (7), Experiment 1 and 2** | | 32 | 19 M, 13 F | | | 4 y/o | | Clear puzzle box with similar mechanism than Horner and Whiten (1) | Stick | | | Stick in Rel and Irrel action | | **Experiment 1**  **% of children copying the irrelevant action**  Unnecessary action paired with correct object: 100 %  Unnecessary action not paired with correct object: 19%  **Experiment 2**  **% of children copying the irrelevant action paired with the object**  Unnecessary action first perinmed by the model beine giving the stick: 0%  Model just give the stick without perinming the irrelevant: 81% | Not indicated | |
| ***Kenward, Karlsson (7), Experiment 3** | | 46 | 30 M, 16 F | | | 5 y/o | | Clear puzzle box with similar mechanism than Horner and Whiten (1) | Stick | | | Stick in Rel and Irrel action | | **% of children saying that they would perinm the irrelevant action**  75% | Not indicated | |
| ***Lyons, Damrosch (8), Experiment 1** | | 64 | Not indicated | | | 4 y/o | | Monkey box (Clear puzzle box) | Wand | | | Wand in Rel and Irrel action | | **% of children copying irrelevant actions**  Non-competitive: 90%  Race 1: 65%  Race 2: 60%  Race 3: 55% | Not indicated | |
| **Lyons, Damrosch (8), Experiment 2** | | 64 | Not indicated | | | 4-5 y/o | | Prize box | No tool | | | Hand in Rel and Irrel action | | **% of children copying irrelevant actions**  Non-competitive: 78%  Competitive: 70% | Not indicated | |
| ***Lyons, Damrosch (8), Experiment 3** | | 27 | Not indicated | | | 3-5 y/o | | Monkey box (Clear puzzle box)  Prize box | Wooden paddle  Wooden wand | | | Wooden paddle in Rel and Irrel actions  Hand in Rel action and wooden wand in Irrel action | | **% of children copying irrelevant actions**  Monkey box: 70%  Prize box: 70% | Not indicated | |
| ***McGuigan, Makinson (9)** | | 24  24  24 | 13 M, 11 F  10 M, 14 F  11, 13 F | | | 3 y/o  5 y/o  Adults | | Clear puzzle box | Stick | | | Stick in Rel and Irrel action | | **Irrelevant tool insertions score (max 1)**  3 y/o: child model: .1,  adult model: .25  5 y/o: child model: .15,  adult model: .5  Adults: child model: .4,  adult model: .75 | Not indicated | |
| **Nielsen and Blank (10)** | | 36 | 19 M, 17 F | | | 4-5 | | Two wooden boxes | No tools | | | Switch Rel action and either swiping on top the lid from right to left three times or taping the right side three times | | **Mean of irrelevant actions (max 3)**  2.50 | Not indicated | |
| ***Flynn and Smith (11), Experiment 1** | | 60 | 6 M, 54 F | | | 25-60 y/o | | Clear puzzle box  Opaque puzzle box | Stick | | | Stick in the Rel and Irrel action | | **% of adults copying irrelevant actions**  Clear: 1^st^ attempt: 95%, 2^nd^ attempt: 95%  Opaque: 1^st^ attempt: 100%, 2^nd^ attempt: 95% | Not indicated | |
| ***Flynn and Smith (11), Experiment 2** | | 32 | 11 M, 21 F | | | 18-39 y/o | | Clear puzzle box  Opaque puzzle box | Stick | | | Stick in the Rel and Irrel action | | **% of adults copying irrelevant actions**  Without demonstrator: Clear: 62.5%, Opaque: 100%  With pressure: Clear: 100%, Opaque: 87.5 % | No | |
| ***Flynn and Smith (11), Experiment 3** | | 32 | 3 M, 29 F | | | 18-29 y/o | | Clear puzzle box  Opaque puzzle box | Stick | | | Stick in the Rel and Irrel action | | **% of adults copying irrelevant actions (faster can win 20 pounds)**  Clear: 68.7%  Opaque: 87.5% | Not indicated | |
| ***Flynn and Smith (11), Experiment 4** | | 32 | 16 M, 16 F | | | 18-21 y/o | | Clear puzzle box  Opaque puzzle box | Stick | | | Stick in the Rel and Irrel action | | **% of adults copying irrelevant actions (model is another participant)**  Clear: 20%  Opaque: 68.7% | No | |
| ***Kenward (12)** | | 24  24 | 16 M, 8 F  14 M, 10 F | | | 3 y/o  4 y/o | | Clear push box  Clear hook box  (similar mechanism than 1) | Pushing stick  Hook stick | | | Pushing stick and hook stick in Rel and Irrel action | | Measures on protest, higher protest when violation of instrumental actions than omitting irrelevant actions | Not indicated | |
| ***McGuigan (13)** | | 44 | 10 M, 34 F | | | 18-25 y/o | | Clear puzzle box  Opaque puzzle box | Stick | | | Stick in Rel and Irrel action | | No percentage or score available in the results, only time to retrieve the reward available | Yes  (towards women due to the model’s sex) | |
| ***Nielsen, Moore (14), Experiment 1** | | 84 | 43 M, 41 F | | | 4 y/o | | Blue box  Switch box  Opaque puzzle box | Red stick  Mallet  Blue stick | | | Stick and mallet in Rel and Irrel action | | **Mean number of irrelevant actions produced**  Condition Prior Experience – Teacher Stays: 2.00  Condition Prior Experience – Student Stays: 1.92  Condition No Experience – Teacher Stays: 2.58  Condition No Experience – Student Stays: 2.08 | No | |
| ***Nielsen, Moore (14), Experiment 2** | | 36 | 18 M, 18 F | | | 4 y/o | | Blue box  Switch box  Opaque puzzle box | Red stick  Mallet  Blue stick | | | Stick and mallet in Rel and Irrel action | | **Mean number of irrelevant actions produced**  Condition Solitary Third Party: 2.17  Condition Social Third Party: 2.50  Condition Direct Modelling: 2.23 | No indicated | |
| **Hilbrink, Sakkalou (15)** | | 37 | Not mentioned | | | 12 and 15 m/o | | Two wooden boxes (containing hidden toy)  Two toy trucks (containing two toys) | No tools | | | Two wooden boxes: remove the Velcro strap (Rel or Irrel action)  Two toy trucks: remove a square cover (Rel or Irrel action) | | **% of children copying the irrelevant action as a first action**  12 months: 46%  15 months: 65% | No | |
| **Keupp, Behne (16)** | | 48  47 | 25 M, 23 F  22 M, 25 F | | | 3 y/o  5 y/o | | Game A  Game B  Game C | Stick  Stick and paintbrush  No tools | | | Open the box in Rel action and stick in Irrel action  Stick in Rel action and paintbrush in Irrel action  Lift barrier in Rel action and turn a clock in Irrel action | | **% of overimitation**  Trial 1: method condition: 3 y/o: 85%, 5 y/o: 90%; goal condition: 3 y/o: 80%, 5 y/o: 70%  Trial 2: method condition: 3 y/o: 75%, 5 y/o: 80%; goal condition: 3 y/o: 70%, 5 y/o: 75% | Not indicated | |
| ***McGuigan (17)** | | 41 | 20 M, 21 F | | | 60-73 m/o | | Clear puzzle box | Stick | | | Stick in Rel and Irrel action | | **Irrelevant action score (max =7)**  Model status: high: 4.5, low: 3 | No | |
| **Nielsen (18)** | | 39 | 19 M, 20 F | | | 4 y/o | | Floating Peanut task | Water | | | Water in Rel and Irrel action | | **% of children copying the irrelevant action**  Small cup condition: 60%  Large cup condition: 64% | Not indicated | |
| **Hoehl, Zettersten (19)** | | 99 | 49 M, 50 F | | | 5 y/o | | Clear puzzle box (different from 1) | Magnetic Rod | | | Rod in Rel and Irrel action | | **% of children copying the irrelevant actions**  Pedagogical then no contact condition: Tapping rod: Phase 1: 28.5%, Phase 2: 14%; Pushing button: Phase 1: 78.6%, Phase 2: 75%;  No contact then pedagogical condition: Tapping rod: Phase 1: 17.8%, Phase 2: 0.07%; Pushing button: Phase 1: 71.4%, Phase 2: 50%;  Pedagogical then pedagogical condition: Tapping rod: Phase 1: 42.8%, Phase 2: 0.07%; Pushing button: Phase 1: 60.7%, Phase 2: 0.25%; | No | |
| **Marsh, Ropar (20)** | | 26  25  22  21 | 10 M, 16 F  17 M, 8 F  10 M, 12 F  10 M, 11 F | | | 5 y/o  6 y/o  7 y/o  8 y/o | | Several boxes and tasks | No tools | | | (e.g., remove elastic band and the lid of the box in the Rel action and slide box along the table in the Irrel action) | | **% of children copying the irrelevant action**  Live demonstration condition: < 6 y/o 7 m/o: 50%, > 6 y/o 7 m/o: 70%  Video demonstration condition: < 6 y/o 7 m/o: 50%, > 6 y/o 7 m/o: 70% | Not indicated | |
| *****†**Nielsen, Mushin (21), Experiment 1** | | 84 Bushman  64 Brisbane | 30 M, 54 F  (equivalent ratio) | | | 3-6 y/o | | Opaque puzzle box  Clear and opaque slab box | Stick | | | Stick in Rel and Irrel action | | **Mean number of irrelevant action (max 7, 5 indicates perfect replication)**  Model/Transfer: Opaque Slab: Brisbane: 4.9, Bushman: 2 ; Clear Slab: Brisbane: 4.8, Bushman: 1.9  Explore/Transfer: Opaque Slab: Brisbane: 1.6, Bushman: 1.9 ; Clear Slab: Brisbane: 1.4, Bushman: 1.1  Model/Same: Opaque Cube: Brisbane: 6.7, Bushman: 5.5 ; Opaque Slab: Brisbane: 6.5, Bushman: 5.8 | Not indicated | |
| *****†**Nielsen, Mushin (21), Experiment 2** | | 19 Indigenous  19 Brisbane | 8 M, 11 F  8 M, 11 F | | | 3-6 y/o | | Opaque puzzle box  Opaque slab box | Stick | | | Stick in Rel and Irrel action | | **Mean number of irrelevant action (max 7, 5 indicates perfect replication)**  Model/Transfer: Phase 1: Indigenous: 5, Brisbane: 2.7; Phase 2: Indigenous: 3.5, Brisbane: 3.7  Model/Experience: Phase 1: Indigenous: 5.2, Brisbane: 3.9; Phase 2: Indigenous: 2.3, Brisbane: 1.7 | Not indicated | |
| *****†**Berl and Hewlett (22)** | | 28 Aka  29 Ngandu  14 Aka adults | 14 M, 14 F  14 M, 15 F  7 M, 7 F | | | 4-7 y/o  20-38 y/o | | Clear puzzle box | Stick | | | Stick in the Rel and Irrel action | | **% of children and adults copying irrelevant action (based on ‘emulation’ graph)**  Aka: 40 %  Ngandu: 85 %  Aka adults: 90 % | No | |
| **Keupp, Behne (23), Experiment 1** | | 32 | 15 M, 17 F | | | 43-65 m/o | | Task A  Task B  Task C  Task D | Stick and feather duster  Paintbrush and long stick  Mechanism integrated in the box  Felt-tipped stick | | | Feather in Rel action and Stick in Irrel action  Stick in Rel action and Paintbrush in Irrel action  Put object in Rel action and turn clock in Irrel action  Put object in Rel action and Stick in Irrel action | | **% of children copying the irrelevant action**  Same context condition: 0 trial: 0, 1 trial: 55%, 2 trials: 45%  Different context condition: 0 trial: 55%, 1 trial: 31%, 2 trials: 14% | Not indicated | |
| **Keupp, Behne (23), Experiment 2** | | 30 | 15 M, 15 F | | | 38-62 m/o | | Task A  Task B  Task C  Task D | Stick and feather duster  Paintbrush and long stick  Mechanism integrated in the box  Felt-tipped stick | | | Feather in Rel action and Stick in Irrel action  Stick in Rel action and Paintbrush in Irrel action  Put object in Rel action and turn clock in Irrel action  Put object in Rel action and Stick in Irrel action | | **% of protest against omission of irrelevant actions**  Same context condition: 47%  Different context condition: 13% | Not indicated | |
| **Keupp, Bancken (24)** | | 57 | 33M, 24 F | | | 4-5 y/o | | Game A  Game B  Game C  Game D | No tools  Stick  No tools  A blue tool | | | Hook the red block in the Rel action and throw a bead in the Irrel action  Use a magnetic stick in the Rel action and rip a sticker and put it onton a yellow board in the Irrel action  Release a marble in the Rel action and rip a sheet paper and put it into a box in the Irrel action  Navigate marble through a labyrinth in the Rel action and junkpress a ball with a blue tool in the Irrel action | | **% of overimitation**  Method: high costs: 40%; low costs: 90%  Goal: hight costs: 20%; low costs: 70% | Not indicated | |
| ***Moraru, Gomez (25)** | | 41  43  57  44 | 20 M, 21 F  20 M, 23 F  33 M, 24 F  22 M, 22 F | | | 3 y/o  4 y/o  5 y/o  6 y/o | | Clear puzzle box | Stick | | | Stick in Rel and Irrel action | | **Mean of irrelevant actions (max 5)**  SS (YT) condition: 3 y/o: 2.8, 4 y/o: 2.0, 5 y/o: 2.5, 6 y/o: 2.5  MS (YT) condition: 3 y/o: 1.3, 4 y/o: 2.2, 5 y/o: 2.5, 6 y/o: 3.4  MS (H) condition: 3 y/o: 1.0 4 y/o: 1.6, 5 y/o: 2.2, 6 y/o: 3.0  MS (H) condition: 3 y/o: 1.5, 4 y/o: 2.6, 5 y/o: 1.6, 6 y/o: 1.9 | No | |
| ***Nielsen, Mushin (26), Experiment 1** | | 48 Brisbane | 28 M, 20 F | | | 3-5 y/o | | Clear puzzle box  Opaque puzzle box | Stick | | | Stick in Rel and Irrel action | | **Mean of overimitation rate (max 7, score 5 represents perfect imitation)**  Brisbane: Single irrelevant: Clear: 5, Opaque: 5.2 ; Pair irrelevant: Clear: 5, Opaque: 4.5 | No | |
| *****†**Nielsen, Mushin (26), Experiment 2** | | 26 Brisbane  26 Borroloola | 15 M, 11 F | | | 3-5 y/o | | Clear puzzle box  Opaque puzzle box | Stick | | | Stick in Rel and Irrel action | | **Mean of overimitation rate (max 7, score 5 represents perfect imitation)**  Brisbane: Clear: 5.5, Opaque: 5.8  Borroloola: Clear: 5, Opaque: 4.3 | No | |
| ***Whiten, Allan (27)** | | 64  64  93 | Not mentioned | | | 4-9 y/o  10-15 y/o  Adults | | Clear puzzle box | Stick | | | Stick in Rel and Irrel action | | **% of participant copying irrelevant actions**  4-9 y/o: 85%  10-15 y/o: 95%  Adults: 70% | Not indicated | |
| **Wood, Harrison (28)** | | 140 | 53 M, 86 F | | | 4-6 y/o | | Slotbox | Rake and arrow tool | | | Each tool perinmed in both Rel and Irrel Action | | **% of children copying the irrelevant actions**  62% | No | |
| **Tanaguchi and Sanefuji (29), Experiment 1** | 16  20  23 | | | 9 M, 7 F  11 M, 9 F  11 M, 9 F | 2 y/o  3 y/o  5 y/o | | Clear puzzle box A  Semi clear and opaque puzzle box B | | | Tool (not indicated) | Tool in both Rel and Irrel action | | **Average numbers of overimitation**  Same apparatus-tool condition: 2 y/o: 0.45, 3 y/o: 0.75, 5 y/o: 0.9  Different apparatus-tool condition: 2 y/o: 0.3, 3 y/o: 0.7, 5 y/o: 0.9  Actor-tool condition: 2 y/o: 0.1, 3 y/o: 0.45, 5 y/o: 0.7 | | | Not indicated |
| **Tanaguchi and Sanefuji (29), Experiment 2** | 24 | | | 14 M, 10 F | 5-6 y/o | | Clear puzzle box A  Semi clear and opaque puzzle box B | | | Tool (not indicated) | Tool in both Rel and Irrel action | | **Average numbers of overimitation**  Same apparatus-tool condition: 0.7  Different apparatus-tool condition: 0.4  Actor-tool condition: 0.35  Actor-no tool condition : 0.2 | | | Not indicated |
| **Vivanti, Hocking (30)** | 31 ASD  18 WS  19 TD | | | 28 M, 3 F  8 M, 10 F  12 M, 7 F | 47 months  51 months  51 months | | Clear box A  Clear box B  Clear box C | | | No tools | A: Push sliders down for Rel action, tap box twice on the slides and lift lids for Irrel actions  B: Pull red handle up for Rel action, push container forward with elbow and turn the lid clockwise for Irrel actions  C: Pull latches up for Rel action, lift contained up and pull lid up for Irrel action | | **Percentage of overimitation**  ASD: 3%  WS: 22%  TD: 37% | | | Not indicated |

**Table S2: Binary logistic linear regression - Success (Pre-demonstration phase) – With all interactions**

| **Predictor** | ***B*** | **S.E.** | **Wald** | ***df*** | ***p*-value** | **Odd-ratio** |
| --- | --- | --- | --- | --- | --- | --- |
| Age | .04 | .02 | 7.40 | 1 | .**007** | 1.05 |
| Cultural background | 1.61 | 2.45 | .43 | 1 | .512 | 4.99 |
| Sex | -4.09 | 3.39 | 1.45 | 1 | .228 | .02 |
| Age*Cultural background | -.01 | .02 | .27 | 1 | .604 | .99 |
| Age*Sex | .03 | .03 | 1.45 | 1 | .228 | 1.04 |
| Cultural background*Sex | -2.39 | 4.56 | .27 | 1 | .600 | .09 |
| Age*Cultural background*Sex | .02 | .04 | .22 | 1 | .636 | 1.02 |

R^2^ = .376 (Nagelkerke); correct classified cases = 78.40%; Model *χ*² (7) = 65.19, *p*-value <.001

**Table S3: Binary logistic linear regression – Success (Post-demonstration) – With all interactions**

| **Predictor** | | ***B*** | **S.E.** | | **Wald** | ***df*** | ***p*-value** | **Odd-ratio** |
| --- | --- | --- | --- | --- | --- | --- | --- | --- |
| Age | | .05 | .04 | | 1.20 | 1 | .274 | 1.05 |
| Cultural background | | .77 | 5.21 | | .02 | 1 | .882 | 2.17 |
| Sex | | .70 | 5.78 | | .01 | 1 | .903 | 2.02 |
| Condition | | -5.44 | 6.80 | | .64 | 1 | .424 | .004 |
| Age*Cultural background | | -.01 | .06 | | .01 | 1 | .906 | .99 |
| Age*Sex | | -.01 | .06 | | .01 | 1 | .929 | .99 |
| Age*Condition | | .07 | .09 | | .58 | 1 | .444 | 1.07 |
| Cultural background*Sex | | 6.49 | 8.77 | | .55 | 1 | .459 | 661.26 |
| Cultural background*Condition | -307.15 | | 41365.97 | | .000 | 1 | .994 | .000 |
| Age*Cultural background*Sex | | .11 | .10 | | 1.18 | 1 | .276 | 1.12 |
| Age*Cultral background*Condition | | 4.96 | 667.19 | | .000 | 1 | .994 | 142.71 |
| Cultural background*Sex*Condition | | -.07 | .11 | | .47 | 1 | .491 | .93 |
| Age*Cultural background*Sex*Condition | | -307.15 | | 41365.97 | .000 | 1 | .994 | .000 |

R^2^ = .36 (Nagelkerke); correct classified cases = 88.70%; Model *χ*² (15) = 29.28, *p*-value = .015

Table S4: Binary logistic linear regression - Over-imitation – With all interactions

| **Predictor** | ***B*** | **S.E.** | **Wald** | ***df*** | ***p*-value** | **Odd-ratio** |
| --- | --- | --- | --- | --- | --- | --- |
| Age | .03 | .02 | 1.82 | 1 | .18 | 1.03 |
| Cultural background | 3.87 | 3.39 | 1.30 | 1 | .25 | 47.76 |
| Sex | 2.07 | 5.87 | .12 | 1 | .72 | 7.95 |
| Age*Cultural background | -.04 | .03 | 1.32 | 1 | .25 | .96 |
| Age*Sex | -.05 | .06 | .55 | 1 | .46 | .95 |
| Cultural background*Sex | -4.83 | 6.77 | .51 | 1 | .48 | .01 |
| Age*Cultural background*Sex | ,06 | .07 | .71 | 1 | .40 | 1.06 |

R^2^ = .24 (Nagelkerke); correct classified cases = 72.5%, Model *χ*² (7) = 14.52, *p*-value = .043

Table S5: Binary logistic linear regression – Success (Overimitation condition) – With all interactions

| **Predictor** | ***B*** | **S.E.** | **Wald** | ***df*** | ***p*-value** | **Odd-ratio** |
| --- | --- | --- | --- | --- | --- | --- |
| Age | .03 | .02 | 1.82 | 1 | .178 | 1.03 |
| Cultural background | 3.28 | 3.39 | .94 | 1 | .333 | 26.62 |
| Sex | 2.07 | 5.87 | .12 | 1 | .724 | 7.95 |
| Age*Cultural background | -.03 | .03 | .81 | 1 | .368 | .97 |
| Age*Sex | -.05 | .06 | .55 | 1 | .460 | .95 |
| Cultural background*Sex | -4.25 | 6.77 | .39 | 1 | .531 | .01 |
| Age*Cultural background*Sex | .05 | .07 | .53 | 1 | .467 | 1.05 |

R^2^ = .26 (Nagelkerke); correct classified cases = 75.00%, Model *χ*² (7) = 16.17, *p*-value = .024

**Table S6: Binary logistic linear regression – Success (Overimitation condition)**

| **Predictor** | ***B*** | **S.E.** | **Wald** | ***df*** | ***p*-value** | **Odd-ratio** |
| --- | --- | --- | --- | --- | --- | --- |
| Age | .06 | .02 | 7.00 | 1 | .008 | 1.06 |
| Cultural background | -.31 | .72 | .19 | 1 | .662 | .73 |
| Sex | -.31 | .72 | .18 | 1 | .672 | .73 |

R^2^ = .225 (Nagelkerke); correct classified cases = 86.00%, Model *χ*² (3) = 10.588, *p*-value = .014

**Table S7: Demographic information regarding (a) the final sample, (b) the French sample and (b) the Serbian sample for the pre-demonstration phase**

1. Demographic information – Final sample

| **Age group (years)** | ***n*** | **Sex** | | ***M*_age_** | ***SD*** | **Age range** |
| --- | --- | --- | --- | --- | --- | --- |
|  |  | **Boy** | **Girl** |  |  |  |
| 5-6 | 52 | 26 | 26 | 6.49 | 0.64 | 5.00-6.92 |
| 7-8 | 66 | 31 | 35 | 8.05 | 0.57 | 7.00-8.92 |
| 9-10 | 57 | 25 | 32 | 10.03 | 0.55 | 9.00-10.92 |
| 11-12 | 33 | 16 | 17 | 11.61 | 0.37 | 11.00-12.33 |
| **Total** | **208** | **98** | **110** | **8.66** | **2.00** | **5.00-12.33** |

1. Demographic information – French sample

| **Age group (years)** | ***n*** | **Sex** | | ***M*_age_** | ***SD*** | **Age range** |
| --- | --- | --- | --- | --- | --- | --- |
|  |  | **Boy** | **Girl** |  |  |  |
| 5-6 | 24 | 14 | 10 | 5.89 | 0.55 | 5.17-6.92 |
| 7-8 | 36 | 17 | 19 | 7.95 | 0.50 | 7.00-8.83 |
| 9-10 | 23 | 9 | 14 | 10.00 | 0.64 | 9.00-10.92 |
| 11-12 | 19 | 7 | 12 | 11.61 | 0.42 | 11.08-12.33 |
| **Total** | **102** | **47** | **55** | **8.61** | **2.08** | **5.17-12.33** |

1. Demographic information – Serbian sample

| **Age group (years)** | ***n*** | **Sex** | | ***M*_age_** | ***SD*** | **Age range** |
| --- | --- | --- | --- | --- | --- | --- |
|  |  | **Boy** | **Girl** |  |  |  |
| 5-6 | 28 | 12 | 16 | 6.22 | 0.98 | 5.00-6.92 |
| 7-8 | 30 | 14 | 16 | 8.17 | 0.62 | 7.00-8.92 |
| 9-10 | 34 | 25 | 32 | 10.05 | 0.50 | 9.00-10.83 |
| 11-12 | 14 | 9 | 5 | 11.53 | 0.42 | 11.08-12.25 |
| **Total** | **106** | **51** | **55** | **8.71** | **1.94** | **5.00;12.25** |

**Table S8: Summary of behaviours observed (frequencies and percentage) in the pre-demonstration phase as a function of age groups and cultural background**

| **Age groups (years)** | **Cultural background** | **Object first entered**  **into the bottle** | | **Kind of pipe cleaner first entered into the bottle** | | **Perseveration** | | **Success** | |
| --- | --- | --- | --- | --- | --- | --- | --- | --- | --- |
|  |  | Pipe cleaner | Other (pipe cleaner + string or string alone) | Pipe cleaner as presented | Pipe cleaner modified | Yes | No | Yes | No |
| 5-6  (*n* = 52) | French  (*n* = 24) | 15  (62.5%) | 9  (37.5%) | 14  (93%) | 1  (7%) | 8  (33%) | 16  (67%) | 2  (8%) | 22  (92%) |
|  | Serbian  (*n* = 28) | 18  (64%) | 10  (36%) | 17  (94%) | 1  (6%) | 14  (50%) | 14  (50%) | 0  (0%) | 28  (100%) |
| **Subtotal** |  | **33**  **(63%)** | **19**  **(37%)** | **31**  **(94%)** | **2**  **(6%)** | **22**  **(42%)** | **30**  **(58%)** | **2**  **(4%)** | **50**  **(96%)** |
| 7-8  (*n* = 66) | French  (*n* = 36) | 28  (78%) | 8  (22%) | 28  (100%) | 0  (0%) | 2  (6%) | 34  (94%) | 6  (17%) | 30  (83%) |
|  | Serbian  (*n* = 30) | 23  (77%) | 7  (23%) | 20  (87%) | 3  (13%) | 6  (20%) | 24  (80%) | 7  (23%) | 23  (77%) |
| **Subtotal** |  | **51**  **(77%)** | **15**  **(23%)** | **48**  **(94%)** | **3**  **(6%)** | **8**  **(12%)** | **58**  **(88%)** | **13**  **(20%)** | **53**  **(80%)** |
| 9-10  (*n* = 57) | French  (*n* = 23) | 20  (87%) | 3  (13%) | 7  (35%) | 13  (65%) | 0  (0%) | 23  (100%) | 14  (61%) | 9  (39%) |
|  | Serbian  (*n* = 34) | 27  (79%) | 7  (21%) | 16  (59%) | 11  (41%) | 5  (15%) | 29  (85%) | 16  (47%) | 18  (53%) |
| **Subtotal** |  | **47**  **(82%)** | **10**  **(18%)** | **23**  **(49%)** | **24**  **(51%)** | **5**  **(9%)** | **52**  **(91%)** | **30**  **(53%)** | **27**  **(47%)** |
| 11-12  (*n* = 33) | French  (*n* = 19) | 19  (100%) | 0  (0%) | 8  (42%) | 11  (59%) | 0  (0%) | 19  (100%) | 13  (68%) | 6  (32%) |
|  | Serbian  (*n* = 14) | 14  (100%) | 0  (0%) | 6  (43%) | 8  (57%) | 3  (21%) | 11  (79%) | 9  (64%) | 5  (36%) |
| **Subtotal** |  | **33**  **(100%)** | **0**  (0%) | **14**  **(42%)** | **19**  **(58%)** | **3**  **(10%)** | **30**  **(90%)** | **22**  **(67%)** | **11**  **(33%)** |
| Total  (*n* = 208) | French  (*n* = 102) | 82  (80%) | 20  (20%) | 57  (70%) | 25  (30%) | 10  (10%) | 92  (90%) | 35  (34%) | 67  (66%) |
|  | Serbian  (*n* = 106) | 82  (77%) | 24  (23%) | 59  (72%) | 23  (28%) | 28  (26%) | 78  (74%) | 32  (30%) | 74  (70%) |
| **All** |  | **164**  **(79%)** | **44**  **(21%)** | **116**  **(71%)** | **48**  **(29%)** | **38**  **(18%)** | **170**  **(82%)** | **67**  **(32%)** | **141**  **(68%)** |

Inter-judge reliability index: κ = .92

**Table S9: Demographic information regarding (a) the final sample, (b) the French sample and (b) the Serbian sample during the post-demonstration phase (Control and Overimitation Condition)**

1. Demographic information – Final sample

| **Control Conditon** | | | | | | | **Overimitation Condition** | | | | | | | | |
| --- | --- | --- | --- | --- | --- | --- | --- | --- | --- | --- | --- | --- | --- | --- | --- |
| **Age group (years)** | ***n*** | **Sex** | | ***M*_age_** | ***SD*** | **Age range** | | ***n*** | **Sex** | | | ***M*_age_** | ***SD*** | **Age range** | |
|  |  | **Boy** | **Girl** |  |  |  |  |  | **Boy** | **Girl** | |  |  |  |  |
| 5-6 | 26 | 14 | 12 | 6.00 | 0.68 | 5.00-6.92 | | 24 | 10 | | 14 | 6.15 | 0.59 | 5.17-6.75 | |
| 7-8 | 19 | 8 | 11 | 8.08 | 0.63 | 7.00-8.75 | | 34 | 14 | | 20 | 8.10 | 0.54 | 7.08-8.75 | |
| 9-12 | 16 | 5 | 1 | 10.26 | 0.90 | 9.00-12.00 | | 22 | 14 | | 8 | 10.39 | 0.88 | 9.17-12.00 | |
| **Total** | **61** | **27** | **34** | **7.76** | **1.89** | **5.00-12.00** | | **80** | **38** | | **42** | **8.14** | **1.75** | **5.17-12.00** | |

1. Demographic information – French sample

| **Control condition** | | | | | | | **Overimitation condition** | | | | | | | |
| --- | --- | --- | --- | --- | --- | --- | --- | --- | --- | --- | --- | --- | --- | --- |
| **Age group (years)** | ***n*** | **Sex** | | ***M*_age_** | ***SD*** | **Age range** | | ***n*** | **Sex** | | | ***M*_age_** | ***SD*** | **Age range** |
|  |  | **Boy** | **Girl** |  |  |  |  |  | **Boy** | **Girl** | |  |  |  |
| 5-6 | 9 | 7 | 2 | 5.66 | 0.41 | 5.17-6.33 | | 13 | 5 | | 8 | 6.02 | 0.55 | 5.17-6.67 |
| 7-8 | 10 | 5 | 5 | 7.95 | 0.49 | 7.08-8.67 | | 20 | 8 | | 12 | 8.04 | 0.50 | 7.08-8.67 |
| 9-12 | 5 | 1 | 4 | 10.10 | 1.04 | 9.17-11.25 | | 10 | 5 | | 5 | 10.56 | 0.91 | 9.25-11.92 |
| **Total** | **24** | **13** | **11** | **7.54** | **1.80** | **5.17-11.25** | | **43** | **18** | | **25** | **8.01** | **1.77** | **5.17-11.92** |

1. Demographic information – Serbian sample

| **Control Conditon** | | | | | | **Overimitation Condition** | | | | | | | | |
| --- | --- | --- | --- | --- | --- | --- | --- | --- | --- | --- | --- | --- | --- | --- |
| **Age group (years)** | ***n*** | **Sex** | | ***M*_age_** | ***SD*** | **Age range** | ***n*** | **Sex** | | | ***M*_age_** | ***SD*** | **Age range** | |
|  |  | **Boy** | **Girl** |  |  |  |  | **Boy** | **Girl** | |  |  |  |  |
| 5-6 | 17 | 7 | 10 | 6.18 | 0.73 | 5.00-6.92 | 11 | 5 | | 6 | 6.29 | 7.51 | 5.17-6.92 | |
| 7-8 | 9 | 3 | 6 | 8.23 | 0.76 | 7.00-8.92 | 14 | 6 | | 8 | 8.18 | 7.34 | 7.08-8.92 | |
| 9-12 | 11 | 4 | 7 | 10.33 | 0.87 | 9.00-12.00 | 12 | 9 | | 3 | 10.26 | 10.45 | 9.17-12.00 | |
| **Total** | **37** | **14** | **23** | **7.91** | **1.95** | **5.00-12.00** | **37** | **20** | | **17** | **8.29** | **1.73** | **5.17-12.00** | |

**Table S10: Frequencies and percentage of touching/using the string and success in the post-demonstration as a function of age groups, cultural background and conditions**

|  |  |  | **Control condition** | | | |  | | | | **Overimitation condition** | | | | | |  | |
| --- | --- | --- | --- | --- | --- | --- | --- | --- | --- | --- | --- | --- | --- | --- | --- | --- | --- | --- |
| **Age Group (years)** | **Cultural background** | | | **Touch/Use of String** | | **Success** | | | **Cultural background** | | | | **Touch/Use of String** | | **Success** | | **Success - Total** | |
|  |  | | | Yes | No | Yes | | No | |  | | Yes | | No | Yes | No | Yes | No |
| 5-6  (*n* = 50)  **Subtotal** | French (*n* = 9) | | | 0  (0) | 9  (100) | 8  (89%) | | 1  (11%) | | French (*n* = 13) | | 2  (15%) | | 11  (85%) | 8  (62%) | 5  (38%) | 16  (73%) | 6  (27%) |
|  | Serbian (*n* = 17) | | | 1  (6%) | 16  (94%) | 13  (76%) | | 4  (23%) | | Serbian (*n* = 11) | | 1  (9%) | | 10  (91%) | 9  (82%) | 2  (8%) | 22  (79%) | 6  (21%) |
|  | **Subtotal** | | | **1**  **(4%)** | **25**  **(96%)** | **21**  **(81%)** | | **5**  **(19%)** | | **Subtotal** | | **3**  **(12.5%)** | | **21**  **(87.5%)** | **17**  **(71%)** | **7**  **(9%)** | **38**  **(76%)** | **12**  **(24%)** |
| 7-8  (*n* = 53)  **Subtotal** | French (*n* = 10) | | | 0  (0%) | 100  (100%) | 10  (100%) | | 0  (0%) | | French (*n* = 20) | | 8  (40%) | | 12  (60%) | 18  (90%) | 2  (10%) | 28  (93%) | 2  (7%) |
|  | Serbian (*n* = 9) | | | 0  (0%) | 9  (100%) | 8  (89%) | | 1  (11%) | | Serbian (*n* = 14) | | 5  (33%) | | 9  (67%) | 13  (93%) | 1  (7%) | 21  (91%) | 2  (9%) |
|  | **Subtotal** | | | **0**  **(0%)** | **19**  **(100%)** | **18**  **(95%)** | | **1**  **(5%)** | | **Subtotal** | | **13**  **(35%)** | | **21**  **(62%)** | **31**  **(91%)** | **3**  **(9%)** | **49**  **(92%)** | **4**  **(8%)** |
| 9-12  (*n* = 38)  **Subtotal** | French (*n* = 5) | | | 0  (0%) | 5  (100%) | 5  (100%) | | 0  (0%) | | French (*n* = 10) | | 4  (40%) | | 6  (60%) | 10  (100%) | 0  (0%) | 15  (100%) | 0  (0%) |
|  | Serbian (*n* = 11) | | | 0  (0%) | 11  (100%) | 11  (100%) | | 0  (0%) | | Serbian (*n* = 12) | | 5  (42%) | | 7  (58%) | 11  (92%) | 1  (8%) | 22  (96%) | 1  (5%) |
|  | **Subtotal** | | | **0**  **(0%)** | **16**  **(100%)** | **16**  **(100%)** | | **0**  **(0%)** | | **Subtotal** | | **9**  **(41%)** | | **13**  **(59%)** | **21**  **(95%)** | **1**  **(5%)** | **37**  **(97%)** | **1**  **(3%)** |
| Total  (*n* = 141)  **All** | French (*n* = 24) | | | 0  (0%) | 24  (100%) | 23  (96%) | | 1  (4%) | | French (*n* = 43) | | 14  (33%) | | 29  (67%) | 36  (84%) | 7  (16%) | 59  (88%) | 8  (12%) |
|  | Serbian (*n* = 37) | | | 1  (3%) | 36  (97%) | 32  (86%) | | 5  (14%) | | Serbian (*n* = 37) | | 11  (30%) | | 26  (70%) | 33  (89%) | 4  (11%) | 65  (88%) | 9  (12%) |
|  | **Subtotal** | | | **1**  **(2%)** | **60**  **(98%)** | **55**  **(90%)** | | **6**  **(10%)** | | **Subtotal** | | **25**  **(31%)** | | **55**  **(69%)** | **69**  **(86%)** | **11**  **(14%)** | **124**  **(88%)** | **17**  **(12%)** |

**Table S11: Frequencies and percentage of overimitators and emulators as a function of age groups, cultural background and sex**

| **Age Group (years)** | **Cultural background** | **Sex** | **Overimitators** | **Emulators** |
| --- | --- | --- | --- | --- |
| 5-6  (*n* = 24) | French (*n* = 13) | Boy (*n* = 5) | 2 (40%) | 3 (60%) |
|  |  | Girl (*n* = 8) | 0 (0%) | 8 (100%) |
|  | *Subtotal French* |  | *2 (15.38%)* | *11 (84.61%)* |
|  | Serbian (*n* = 11) | Boy (*n* = 6) | 0 (0%) | 6 (100%) |
|  |  | Girl (*n* = 5) | 1 (20%) | 4 (80%) |
|  | *Subtotal Serbian* |  | *1 (9.09%)* | *10 (90.91%)* |
|  |  | ***Subtotal Boy (n = 11)*** | ***2 (18.18%)*** | ***9 (81.82%)*** |
|  |  | ***Subtotal Girl (n = 13)*** | ***1 (7.69%)*** | ***12 (92.31%)*** |
| **Subtotal** |  |  | **3 (12.5%)** | **21 (87.5%)** |
| 7-8  (*n* = 34) | French (*n* = 20) | Boy (*n* = 8) | 4 (50%) | 4 (50%) |
|  |  | Girl (*n* = 12) | 3 (25%) | 9 (75%) |
|  | *Subtotal French* |  | *7 (35%)* | *13 (65%)* |
|  | Serbian (*n* = 14) | Boy (*n* = 6) | 3 (50%) | 3 (50%) |
|  |  | Girl (*n* = 8) | 1 (12.5%) | 7 (87.5%) |
|  | *Subtotal Serbian* |  | *4 (28.57%)* | *10 (71.43%)* |
|  |  | ***Subtotal Boy (n = 14)*** | ***7* (50%)** | ***7 (50%)*** |
|  |  | ***Subtotal Girl (n = 20)*** | ***4 (20%)*** | ***16 (80%)*** |
| **Subtotal** |  |  | **11 (32.35%)** | **23 (67.65%)** |
| 9-12  (*n* = 22) | French (*n* = 10) | Boy (*n* = 5) | 2 (40%) | 3 (60%) |
|  |  | Girl (*n* = 5) | 1 (33%) | 4 (67%) |
|  | *Subtotal French* |  | *3 (30%)* | *7 (70%)* |
|  | Serbian (*n* = 12) | Boy (*n* = 9) | 5 (42.86%) | 4 (57.14%) |
|  |  | Girl (*n* = 3) | 0 (0%) | 3 (100%) |
|  | *Subtotal Serbian* |  | *5 (33.34%)* | *7 (66.66%)* |
|  |  | ***Subtotal Boy (n = 14)*** | ***7 (50%)*** | ***7 (50%)*** |
|  |  | ***Subtotal Girl (n = 8)*** | ***1 (20%)*** | ***7 (80%)*** |
| **Subtotal** |  |  | **8 (36.36%)** | **14 (63.64%)** |
| Total  (*n* = 80) | French (*n* = 43) | Boy (*n* = 18) | 8 (44.45 %) | 10 (55.55%) |
|  |  | Girl (*n* = 25) | 4 (16%) | 21 (84%) |
|  | *Subtotal French* |  | 13 (30.23/%) | 30 (69.77%) |
|  | Serbian (*n* = 37) | Boy (*n* = 20) | 9 (45%) | 11 (55%) |
|  |  | Girl (*n* = 17) | 1 (5.88%) | 16 (94.12%) |
|  | *Subtotal Serbian* |  | 10 (27.03%) | 27 (72.97%) |
|  |  | ***Subtotal Boy (n = 38)*** | ***17 (44.74%)*** | ***21 (55.26%)*** |
|  |  | ***Subtotal Girl (n = 42)*** | ***5 (11.9%)*** | ***37 (88.1%)*** |
| **All** |  |  | **22 (27.5%)** | **58 (72.5%)** |

**References**

1. Horner V, Whiten A. 2005 Causal knowledge and imitation/emulation switching in chimpanzees (Pan troglodytes) and children (Homo sapiens). *Anim. Cogn*. **8**, 164-81.

2. Lyons DE, Young AG, Keil FC. 2007 The hidden structure of overimitation. *Proc. Natl. Acad. Sci. U.S.A*. **104**, 19751-19756.

3. McGuigan N, Whiten A, Flynn E, Horner V. 2007 Imitation of causally opaque versus causally transparent tool use by 3-and 5-year-old children. *Cognitive Dev*. **22**, 353-64.

4. McGuigan N, Whiten A. 2009 Emulation and “overemulation” in the social learning of causally opaque versus causally transparent tool use by 23-and 30-month-olds. *J. Exp. Child Psychol*. **104**, 367-81.

5. Nielsen M, Tomaselli K. 2010 Overimitation in Kalahari Bushman children and the origins of human cultural cognition. *Psychol Sci*. **21**, 729-36.

6. McGuigan N, Graham M. 2010 Cultural transmission of irrelevant tool actions in diffusion chains of 3-and 5-year-old children. *Eur. J. Dev.Psychol*. **7**,561-77.

7. Kenward B, Karlsson M, Persson J. 2011 Over-imitation is better explained by norm learning than by distorted causal learning. *Proc. R. Soc. B* **278**, 1239-46.

8. Lyons DE, Damrosch DH, Lin JK, Macris DM, Keil FC. 2011 The scope and limits of overimitation in the transmission of artefact culture. *Phil. Trans. R. Soc. B* **366**, 1158-67.

9. McGuigan N, Makinson J, Whiten A. 2011 From over‐imitation to super‐copying: Adults imitate causally irrelevant aspects of tool use with higher fidelity than young children. *Br. J. Psychol.* **102**, 1-18.

10. Nielsen M, Blank C. 2011 Imitation in young children: When who gets copied is more important than what gets copied. *Dev. Psychol*. **47**, 1050.

11. Flynn S, Smith, K. 2012 Investigating the mechanisms of cultural acquisition: How pervasive is overimitation in adults? *Soc. Psychol*. **43**, 185.

12. Kenward B. 2012 Over-imitating preschoolers believe unnecessary actions are

normative and enforce their performance by a third party. *J. Exp. Child Psychol*. **112**,

195-207.

13. McGuigan N. 2012 The role of transmission biases in the cultural diffusion of irrelevant actions. *J. Comp. Psychol*. *126*, 150.

14. Nielsen M, Moore C, Mohamedally J. 2012 Young children overimitate in third-party contexts. *J. Exp. Child Psychol*. **112**, 73-83.

15. Hilbrink EE, Sakkalou E, Ellis‐Davies K, Fowler NC, Gattis M. 2013 Selective and faithful imitation at 12 and 15 months. Dev. Sci. **16**, 828-40.

16. Keupp S, Behne T, Rakoczy H. 2013 Why do children overimitate? Normativity is crucial. *J. Exp. Child Psychol*. **116**, 392-406.

17. McGuigan N. 2013 The influence of model status on the tendency of young children to over-imitate. *J. Exp. Child Psychol*. **116**, 962-969.

18. Nielsen M. 2013 Young children's imitative and innovative behaviour on the floating object task. *Infant Child Dev*. **22**, 44-52.

19. Hoehl S, Zettersten M, Schleihauf H, Grätz S, Pauen S. 2014 The role of social interaction and pedagogical cues for eliciting and reducing overimitation in preschoolers. *J. Exp. Child Psychol*. **122**, 122-33.

20. Marsh LE, Ropar D, Hamilton AFDC. 2014 The social modulation of imitation fidelity in school-age children. *PLOS ONE*. **9**, e86127.

21. Nielsen M, Mushin I, Tomaselli K, Whiten A. 2014 Where culture takes hold:“Overimitation” and its flexible deployment in Western, Aboriginal, and Bushmen children. *Child Dev.* **85**, 2169-84.

22. Berl RE, Hewlett BS. 2015 Cultural variation in the use of overimitation by the Aka and Ngandu of the Congo Basin. *PLOS ONE* **10**, e0120180.

23. Keupp S, Behne T, Zachow J, Kasbohm A, Rakoczy H. 2015 Over-imitation is not automatic: Context sensitivity in children’s overimitation and action interpretation of causally irrelevant actions. *J. Exp. Child Psychol*. **130**, 163-75.

24. Keupp S, Bancken C, Schillmöller J, Rakoczy H, Behne T. 2016 Rational over-imitation: Preschoolers consider material costs and copy causally irrelevant actions selectively. *Cognition* ***147***, 85-92.

25. Moraru C-A, Gomez J-C, McGuigan N. 2016 Developmental changes in the influence of conventional and instrumental cues on over-imitation in 3-to 6-year-old children. *J. Exp. Child Psychol*. **145**, 34-47.

26. Nielsen M, Mushin I, Tomaselli K, Whiten A. 2016 Imitation, Collaboration, and Their Interaction Among Western and Indigenous Australian Preschool Children. *Child Dev*. 87, 795-806.

27. Whiten A, Allan G, Devlin S, Kseib N, Raw N, McGuigan N. 2016 Social Learning in the Real-World:‘Over-Imitation’Occurs in Both Children and Adults Unaware of Participation in an Experiment and Independently of Social Interaction. *PLOS ONE* **11**, e0159920.

28. Wood LA, Harrison RA, Lucas AJ, McGuigan N, Burdett ER, Whiten A. 2016 “Model age-based” and “copy when uncertain” biases in children’s social learning of a novel task. *J. Exp. Child Psychol*. **150**, 272-84.

29. Taniguchi Y, Sanefuji W. 2017 The boundaries of overimitation in preschool

children: Effects of target and tool use on imitation of irrelevant actions. *J. Exp. Child*

*Psychol*. **159**, 83-95.

30.Vivanti G, Hocking DR, Fanning P, Dissanayake C. 2017 The social nature of

overimitation: Insights from Autism and Williams syndrome. *Cognition*, **161**, 10-18.
